# Scale-Up and Long-Term Study of Electrodialysis with Ultrafiltration Membrane for the Separation of a Herring Milt Hydrolysate

**DOI:** 10.3390/membranes11080558

**Published:** 2021-07-23

**Authors:** Jacinthe Thibodeau, Noémie Benoit, Véronique Perreault, Laurent Bazinet

**Affiliations:** Laboratoire de Tranformation Alimentaire et Procédés ÉlectroMembranaires (LTAPEM, Laboratory of Food Processing and Electromembrane Process), Department of Food Sciences, Institute of Nutrition and Functional Foods (INAF), Université Laval, Québec, QC G1V 0A6, Canada; Jacinthe.thibodeau.1@ulaval.ca (J.T.); Noemie.benoit.1@ulaval.ca (N.B.); Veronique.perreault.5@ulaval.ca (V.P.)

**Keywords:** electrodialysis, filtration membrane, herring milt hydrolysate, peptide migration rate, energy consumption, global system resistance, membrane fouling

## Abstract

Electrodialysis with ultrafiltration membrane (EDUF) was selected to separate a herring milt hydrolysate (HMH) in a scale-up and long-term study for the recovery of bioactive peptides. The scale-up was performed to maximise peptide recovery by placing a total membrane area of 0.08 m^2^ for each anionic and cationic compartment. Twelve consecutive runs were carried out, for a total of 69 h, with minimal salt solution cleaning in between experiments. The final peptide migration rate showed that cationic peptides had a higher average migration rate (5.2 ± 0.8 g/m^2^·h), compared to anionic peptides (4.7 ± 1.1 g/m^2^·h). Migration was also selective according to peptide identifications and molecular mass distribution where only small molecular weights were found (<1000 Da) in both recovery compartments. The areal system resistance slightly decreased during each run and the averaged values were stable in between experiments since they were all found in the 95% confidence interval. In addition, total relative energy consumption was quite consistent with an average value of 39.95 ± 6.47 Wh/g all along the 12 consecutive runs. Finally, according to membrane characterization, there was no visual fouling on the different membranes present in the EDUF cell after 69 h of treatment. This may be due to the salt cleaning in between experiments which allowed removal of peptides from the membranes, thus allowing recovering initial system working parameters at the beginning of each run. The entire process was revealed to be very consistent and repeatable in terms of peptide migration, global system resistance, and energy consumption. To the best of our knowledge, this is the first time such EDUF conditions (membrane surface, duration, and minimal salt cleaning between experiments) are being tested on a complex hydrolysate.

## 1. Introduction

In the past decades, electromembrane processes have widely been used to separate and concentrate bioactive peptides and to valorise by-products in the agri-food industry [1,2]. Electrodialysis (ED), which uses an electrical field as the driving force, is an eco-efficient technology known to effectively separate molecules for different applications such as demineralisation, stabilisation, separation, purification, and deacidification purposes [3,4]. More specifically, electrodialysis with ultrafiltration membrane (EDUF) is a technology of choice for the separation of bioactive molecules since it can simultaneously separate molecules by both their charge and size. Dlask and Václavíková [5] reported different types of hydrolysates that have been fractionated by EDUF in the past years (beta-lactoglobulin, soy, flaxseed, snow crab and, more recently, spent yeasts [6]) for the recuperation of a variety of bioactive peptides having a huge potential to be used as functional foods. Therefore, in order to produce large amounts of bioactive peptides from a hydrolysate, the development of an industrial scale EDUF process is necessary [7].

Many studies have demonstrated the feasibility of this process at laboratory scale by using EDUF stacks with an effective surface area from 10 to 40 cm^2^, by stacking 1 to 4 ultrafiltration (UF) membranes on a microflow type cell [8] to 100 cm^2^, or on a MP type cell [9,10]. Recently, at lab scale, Henaux et al. [10] used three UF membrane cut-offs (50, 20, and 5 kDa) for the separation of a salmon protein hydrolysate. They were able to achieve a maximum peptide migration rate of 3.19 ± 0.14 g/m^2^·h for the cationic recovery compartment, and that configuration generated a relative energy consumption (REC) of 512.56 ± 95.59 Wh/g. Additionally, the molecular weight distribution of the peptide profiles revealed molecular weights lower than 1000 Da with a maximum between 300–500 Da. On the other hand, Durand et al. [11,12], also at lab scale, worked with two herring milt hydrolysates (one which was the same as in this study, namely, PG1), but with a double simultaneous separation (2 UF membranes of 20 and 50 kDa for cationic and anionic peptide recovery). The highest peptide migration rate obtained was also with the cationic 50 kDa recovery compartment with a value of 3.12 ± 0.55 g/m^2^·h for initial hydrolysate and 8.79 ± 0.24 g/m^2^·h for PG1. For initial hydrolysate, some molecular masses over 10 kDa were reported, but mainly the molecular mass distribution displays masses lower than 700 Da in the different recovery compartments. Finally, the REC obtained for the entire stack, for initial hydrolysate (not mentioned for PG1) was 46.99 ± 6.59 Wh/g.

In the context of scaling up the EDUF process, and to the best of our knowledge, only four studies concerning scale-up of EDUF at pilot scale (EUR2 cell) with membrane surface area ≥ 200 cm^2^ are reported in the literature (Table 1). Firdaous et al. [13] carried out, for the first time, experiments on a EUR2-C pilot-scale developing a membrane area of 0.12 m^2^ by stacking 6 UF membranes for the recovery of cationic peptides in a first configuration (Cationic configuration) and anionic peptides in a second configuration (Anionic configuration) from alfalfa white protein hydrolysate (0.5% (*w*/*v*)). For the cationic configuration, the average transport rate of peptides calculated during the 120 min of EDUF was 5.3 g/m^2^·h, while for the anionic configuration, it was 8.7 g/m^2^·h. Doyen et al. [14] also carried out experiments, for the simultaneous recovery of anionic and cationic peptides, with a snow crab hydrolysate (1% *v*/*v*) on a EUR2-C pilot-scale system by stacking 6 UF membranes, to reach a total membrane surface of 0.06 m^2^ per recovery compartment. Finally, Langevin et al. [15] and Roblet et al. [16] both worked on a soy protein hydrolysate for the separation of bioactive peptides with a EUR2-C cell. The main differences in both studies are that Langevin et al. [15] used four cells to obtain an effective membrane area of 0.08 m^2^ per recovery compartment, compared to the configuration of Roblet et al. [16] which had only three cells (0.06 m^2^). Additionally, Langevin et al. [15] worked with a low hydrolysate concentration of 0.1% (*w*/*v*), compared to 2.4% (*w*/*v*) for Roblet et al. [16]. These conditions were reflected in the peptide migration rates obtained by Langevin et al. [15] which were very low, varying from 0.109 to 0.165 g/m^2^·h for the anionic recovery compartment and from 0.238 to 0.097 g/m^2^·h for cationic recovery compartment. On the other hand, conditions reflected in Roblet et al. [16] allowed obtaining higher final migration rates of 1.3 g/m^2^·h and 2.4 g/m^2^·h for anionic peptides and cationic peptides, respectively (Table 1). These values can be explained by the hydrolysate concentration since one extra cell of membranes in the stack was not enough to favour higher peptide migration. Moreover, cell configuration is a very important factor as Firdaous et al. [13] showed a higher peptide migration rate by testing either cationic or anionic peptide recovery one at a time. Hence, to evaluate the performances of the EDUF process and whether those findings at bench scale translate to pilot scale, after selecting the proper configuration type and hydrolysate starting concentration, it is important to analyse the main metrics of this hybrid technology, different from both conventional ED and ultrafiltration, such as peptide migration rates, molecular weight distribution of recovered peptides, global system resistance, energy consumption, and membrane fouling.

Since studies over 12 h in the same condition with minimum CIP (Table 1) have never been carried out to attest the repeatability and reproducibility of EDUF on a complex hydrolysate, the specific objectives of the present work were to (1) assess the long-term feasibility of EDUF process at pilot scale during consecutive runs, (2) evaluate the performances of the process in terms of global system resistance, energy consumption, and peptide migration rates, (3) evaluate molecular weight distribution of recovered fractions and selectivity, and (4) outline membrane fouling.

## 2. Materials and Methods

### 2.1. Materials

#### 2.1.1. Chemicals

Chemicals used to prepare solutions for the EDUF process were either purchased from Fisher Scientific (Montréal, QC, Canada) for hydrochloric acid (HCl) and sodium hydroxide (NaOH) or BDH (VWR International Inc., Mississauga, ON, Canada) for sodium sulfate (Na_2_SO_4_), potassium chloride (KCl) and sodium chloride (NaCl).

#### 2.1.2. Herring Milt Hydrolysate

The herring milt hydrolysate (HMH) (commercially named PG1) was provided by Ocean NutraSciences Inc. (Matane, QC, Canada). It consists of hydrolysed herring milt which is ultrafiltered and the recovered permeate is, afterwards, spray-dried to obtain a fine powder (confidential process). The chemical composition of the initial hydrolysate (IH) was: 97.73 ± 0.44% of total nitrogen, 93.79 ± 0.46% of peptide, 7.33 ± 0.49% of nucleic acids, 12.28 ± 0.50% ashes, and 3.85 ± 0.32% of humidity, as well, for amino acid composition, arginine had a value of 15.27 ± 0.99 g/100 g peptide [11].

### 2.2. Electrodialysis System and Working Parameters

**Figure 1:**
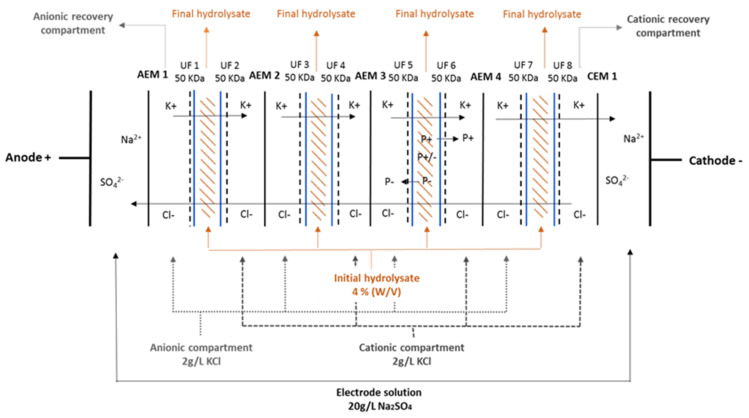
EDUF configuration used for the simultaneous separation of the herring milt hydrolysate: P+/− neutral peptides, P+ cationic peptides, P− anionic peptides, blue line: filter side of UF membranes, dotted line: other side of the UF membranes.

The ED system used for these scale-up experiments was a semi-pilot scale system composed of three 10 L compartments for recovery of the peptides and hydrolysate and one 4 L compartment for electrode rinsing solution. The solutions were circulated using centrifugal pumps (Baldor Electric Co., Fort Smith, AR, USA), and the flow rates were controlled by flow meters (Aalborg Instruments and Controls, Inc., Orangeburg, SC, USA). The module selected for this scale-up was a EUR2-C (Ameridia Innovative Solutions Inc., CA, USA) with an effective membrane surface area of 200 cm^2^ (Table 1). Both electrodes were dimensionally stable electrodes (DSE). Eight UF membranes (50 kDa polyethersulfone (PES), Synder Filtration, Vacaville, CA, USA), four Neosepta anion-exchange membranes (AEM), and one Neosepta cation-exchange membrane (CEM), both manufactured by Astom (Tokyo, Japan), but provided by Ameridia Innovative Solutions Inc. (CA, USA) were stacked in between the electrodes (Figure 1). The configuration consisted of two electrode compartments (Na_2_SO_4_ 20 g/L, 3 L), four cationic peptide recovery compartments (C^+^RC, KCl 2 g/L, 3 L), four anionic recovery compartments (A^−^RC, KCl 2 g/L, 3 L), and four HMH compartments (3 L). Briefly, a 4% (*w*/*v*) isolate was prepared at pH 7 (natural pH of the hydrolysate) and left for solubilisation overnight with agitation at 10 °C. The next day, all solutions were poured into their respective reservoirs. Parameters were adjusted (pH, conductivity), and then a constant voltage (14 V) was applied to have constant electrical field strength for the separation of this herring milt hydrolysate [15]. This value was chosen regarding the previous study by Durand et al. [11] with minor adjustments due to the scale of the system and stack used. Electric current (measured directly on the power supply (Model HPD 30–10, Xantrex, Burnaby, BC, Canada), conductivity, and pH values were measured each 30 min during the 4–6 h treatment. The pH and conductivity values were maintained constant during the experiments to allow continuous peptide migration [9]. For pH, solutions of either HCl or NaOH at 1.0 M were added when necessary; as for conductivity, KCl was added in the reservoir when needed. Samples were taken after 30 min, 60 min, and after each hour afterward. Samples were kept at −20 °C until further analyses (determination of peptide migration rate, final peptide concentration and their identification and determination of relative molecular weight distribution by mass spectrometry). A minimal cleaning procedure was performed on the system after each experiment using NaCl 2% solution [17,18]. Even if chlorine gas formation can occur in the electrolyte rinsing compartment, it should not affect the ionic membrane performances since, with the knowledge from our industrial partners, the duration of the process is not long enough; thus, this parameter becomes negligible. Finally, the cell was rinsed with water and kept in KCl 2 g/L overnight until another experiment was carried out. A total of 12 experiments, for 69 h of total process time, were conducted: the first experiment lasted 4 h because it was a test run, runs 2 to 11 lasted 6 h, and experiment 12 lasted 5 h due to technical changes. At the end of the 12 experiments, the stack was dismantled, and membranes were characterized for their thickness and conductivity to determine if any fouling occurred.

### 2.3. Methods

#### 2.3.1. pH

pH was measured during EDUF treatments using a pH-meter model SP20 (Thermo Orion, West Chester, PA, USA) equipped with a VWR Symphony epoxy gel combination pH electrode (Montréal, QC, Canada).

#### 2.3.2. Conductivity

Conductivity was monitored during EDUF experiments using an YSI conductivity meter (Model 3100) equipped with an YSI immersion probe model 3252, cell constant K = 1 cm^−1^ (Yellow Springs Instrument CO., Yellow Springs, OH, USA).

#### 2.3.3. Peptide Migration in Recovery Compartments and Final Migration Rate

The peptide concentration was measured in liquid samples taken in both recovery compartments over time using the Pierce^®^ BCA Protein Assay supplied from Fisher (Montréal, QC, Canada). Briefly, 25 μL of the non-diluted sample was mixed with 200 μL of working reagent into a 96-well microplate and incubated at 37 °C for 30 min. The absorbance was then read at 562 nm using a spectrophotometer (xMark Microplate spectrophotometer, Bio-Rad, Mississauga, ON, Canada). On the other hand, the total nitrogen content was determined on the spray-dried powders by the Dumas combustion method using a Rapid Micro N Cube (Elementar, Francfort-sur-le-Main, Germany) and was converted into a protein concentration by applying a 6.25 conversion factor to obtain the final peptide quantity in grams.

The final migration rate (g /m^2^·h) corresponds to the final peptide quantity (g) divided by the ultrafiltration membrane surface (0.08 m^2^) and by the duration of separation (4, 5, or 6 h, depending on the experiment) (Equation (1)).

(1) $$\mathrm{Final}\;\mathrm{migration}\;\mathrm{rate}=\frac{\mathrm{peptide}\;\left(g\right)}{\mathrm{membrane}\;\mathrm{surface}\;\left(m^{2}\right)*\mathrm{time}\;\left(h\right)}$$

#### 2.3.4. Identification of Peptides in Recovery Compartments

RP-UPLC analyses were performed using a 1290 Infinity II UPLC (Agilent Technologies, Santa Clara, CA, USA). The equipment was composed of a binary pump (G7120A), a multisampler (G7167B), an in-line degasser, and a variable wavelength detector (VWD G7114B) adjusted to 214 nm. Peptide samples were prepared at a 0.5% (5 mg/mL) concentration and were filtered through a 0.22 μm PVDF filter into a glass vial. They were, afterward, loaded (10 μL) onto an Acquity UPLC CSH 130 1.7 μm C18 column (2.1 × 150 mm i.d., Waters Corporation, Milford, MA, USA) with the following operating parameters: flow rate of 0.4 mL/min and temperature of 45 °C. A linear gradient consisting of solvent A (LC-MS grade water with 0.1% formic acid) and solvent B (LC-MS grade acetonitrile with 0.1% formic acid) was applied with solvent B increasing from 2% to 25% in 50 min holding until 54 min after ramping to 90% and then back to initial conditions. A hybrid ion mobility quadrupole time of flight mass spectrometer (model 6560 IM-Q-TOF, Agilent, Santa Clara, CA, USA) was used to identify the peptides present in the hydrolysate. The entire UPLC-MS/MS experiments were acquired using Q-TOF. Signals were recorded in positive mode at Extended Dynamic Range, 2 GHz, 3200 m/z with a scan range between 100 and 3200 m/z. Nitrogen was used as the drying gas (13.0 L/min and 150 °C) and as nebuliser gas (30 psi). The capillary voltage was set at 3500 V, the nozzle voltage at 300 V, and the fragmentor at 400 V. The instrument was calibrated using an ESI-L low concentration tuning mix (Agilent Technologies, Santa Clara, CA, USA). Data acquisition and analysis were performed using the Agilent Mass Hunter Software package (LC/MS Data Acquisition, Version B.08.00 and Qualitative Analysis for IM-MS, Version B.07.00 Service Pack 2 with BioConfirm Software). An additional search was conducted using the Spectrum Mill MS Proteomics Workbench (Rev B.05.00.180). The *Clupea harengus* NCBI protein database was used to search and identify peptides.

#### 2.3.5. Areal System Resistance and Relative Energy Consumption

The areal system resistance was calculated according to Ohm’s law (R = V/A). The voltage (Volts (V)) and electric current (Amps (A)) values were directly obtained from the power supply and, finally, this value in Ohm was divided by the electrode surface (200 cm^2^).

The relative energy consumption was calculated using the following equation (Equation (2)):

(2) $$E=\frac{U.I.\Delta \;t}{3600*g\;\mathrm{of}\;\mathrm{peptides}}$$

where E is the relative energy consumption in Wh/g of migrated peptides, U the voltage applied (in V), I electric current (in A) measured, Δ t time interval between values (in s) taken during the run.

#### 2.3.6. Membrane Thickness

The different membranes were characterized before and after the entire 12 experiments by measuring their thickness with an electronic digital micrometer (Marathon Watch Company Ltd., Richmond Hill, ON, Canada).

#### 2.3.7. Membrane Electrical Conductivity

Each membrane was characterized before and after the 12 experiments by determining their conductivity with a specially designed clip from Laboratoire des Matériaux Échangeurs d’Ions (Université Paris XII, Creteil, Val de Marne, France). The equation used to calculate these values is the following (Equation (3)) [19,20]:

(3) $$\kappa =\frac{l}{R_{m}*A}$$

where ĸ = membrane conductivity (mS/cm), R_m_ = membrane resistance (mΩ), A = electrodes area = 1 cm^2^ and l = thickness of the membrane (cm).

#### 2.3.8. Statistical Analyses

For statistical analyses on membrane characterization, t-tests were performed at a probability level of *p* ≤ 0.05. They were used to compare membranes before and after treatment for fouling determination (Sigma Plot Software, Version 14.0 for Windows, Systat Software Inc., San Jose, CA, USA). T-tests were also conducted for peptide migration, final migration rate, and relative energy consumption to compare between cationic and anionic recovery compartments. For global system resistance, regressions were calculated. Finally, for the average global system resistance and relative energy consumption, confidence intervals and regressions were calculated.

## 3. Results and Discussion

### 3.1. Peptide Migration in Recovery Compartments and Final Migration Rate

**Figure 2:**
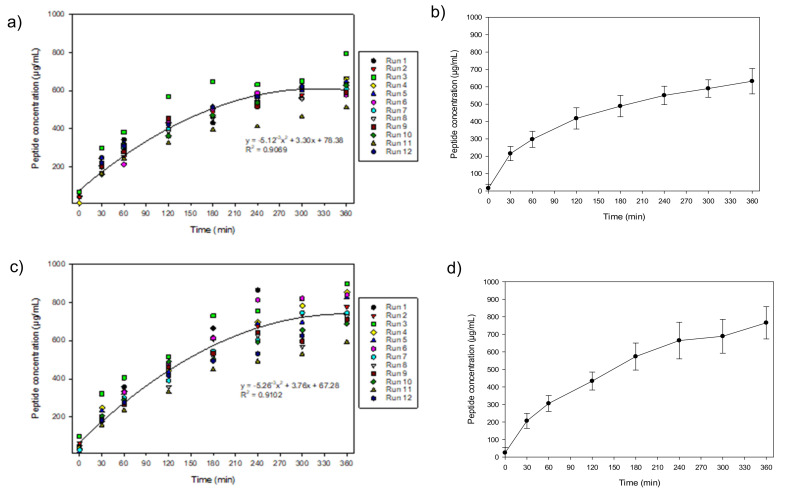
Migration rate of peptides for the 12 consecutive experiments in the (**a**) cationic recovery compartment (C^+^RC) and (**c**) anionic recovery compartment (A^−^RC) and average migration rate for (**b**) cationic recovery compartment (C^+^RC) and (**d**) anionic recovery compartment (A^−^RC).

**Figure 3:**
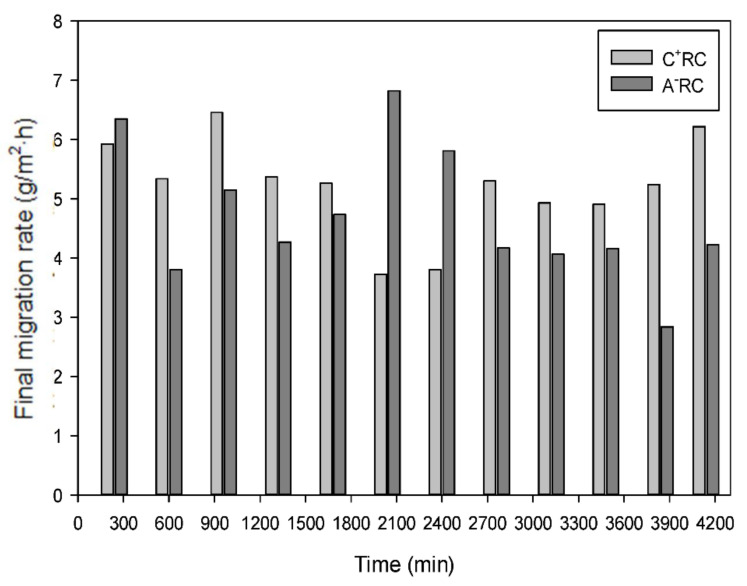
Final migration rate for both recovery compartments after each consecutive EDUF run for a total process time of 4140 min (69 h).

After 300 min of treatment, it was observed that the migration rate of cationic peptides in C^+^RC slowed down and seemed to reach a plateau (Figure 2a, R^2^ = 0.9069), while for anionic peptide’s migration in A^−^RC (Figure 2c, R^2^ = 0.9102), even after 360 min of treatment, a plateau was not reached. It is important to mention that the main parameters impacting the EDUF process (pH, conductivity, and voltage/electric field strength) were maintained constant all along the process explaining the good repeatability of the 12 experiments (high R^2^ values of the regression curves). The difference observed for some repetitions should be due to less effective cleaning of the membranes. If needed, the process could have lasted longer to recover more anionic peptides. The averaged curves of peptide migration (Figure 2b,d) illustrate that trend. Thus, the *p*-value obtained (*p* = 0.0007) demonstrated, with a statistical difference, that more anionic than cationic peptides were recovered after 360 min with an average of 762.44 ± 97.97 µg/mL and 626.70 ± 68.17 µg/mL, respectively. On the other hand, the final migration rate showed no significant difference (*p* = 0.226) for cationic peptides with an average of 5.2 ± 0.8 g/m^2^·h, compared to anionic peptides with 4.7 ± 1.1 g/m^2^·h after a total process time of 4140 min (69 h) (Figure 3 and Table 1). The peptide migration is calculated based on liquid samples taken in the compartments during the EDUF experiments, while the final migration rate is obtained after spray-drying of the samples, on a dry powder basis. In order to better follow peptide migration, the percentage of peptides that were actually separated in the recovery compartments, compared to the starting concentration of the hydrolysate was determined. Considering the 4% initial concentration (120 g of peptides in 3 L), an average of only 4.52 g of peptides was separated in the cationic and anionic compartments combined, which corresponds to 3.77% of total peptides in the HMH. This means that a large proportion of peptides are still available for migration, but that the recovered fraction may be concentrated in bioactive peptides, and the specificity of the peptides separated is generally really high in comparison with other processes [4,10,11,15]. Usually, the separation of anionic from cationic peptides and from initial hydrolysate allows separating bioactive peptides from inhibitory ones (Durand et al. [12]). It all depends on the type of hydrolysate since each hydrolysate is different and needs to be studied and bioactivities tested. Nevertheless, according to the previous study by Durand et al. [11], on the same HMH with a 100 cm^2^ MP type cell, it was also observed that the final migration rate was higher for the cationic recovery compartment than for the anionic recovery compartment (8.79 ± 0.24 vs. 8.35 ± 0.5 g/m^2^·h) even if there was a very low percentage of peptides that were separated (0.59%) due to the four compartments configuration and duration of the process. The result obtained in this study is in accordance with this previous study which is encouraging since the same hydrolysate was used for scaling-up. However, an important parameter that may also explain these results is the pH of the hydrolysate. In their Supplementary Materials Section, Durand et al. [11] clearly demonstrated the effect of pH of the HMH on peptide migration in EDUF. It was observed that, depending on the pH of the hydrolysate, the charges of the peptides would vary, mainly due to their isoelectric point (pI). For example, at pH 3, more cationic peptides were obtained with a final migration rate of 12.60 ± 1.14 g/m^2^·h vs. 2.36 ± 0.45 g/m^2^·h for anionic peptides. The opposite trend was observed for pH 9 (14.64 ± 0.95 g/m^2^·h for anionic and 7.81 ± 0.68 g/m^2^·h for cationic), and values were more similar at pH 6 with 10.81 ± 0.28 g/m^2^·h and 11.49 ± 2.51 g/m^2^ h for anionic and cationic recovery compartments, respectively. Amino acid analysis of the initial HMH, adjusted at pH 7 for the EDUF experiments, also carried out by Durand et al. [11], demonstrated that the amount of positively charged amino acids present, such as arginine (Arg), was higher than negatively charged amino acids. Indeed, the fact that the peptides in the initial HMH of this study are mainly composed of Arg, a basic amino acid whose pI is at 10.76, would explain why more cationic peptides were recovered even at pH 7. Since the HMH in this study was in the same conditions as Durand et al. [11], it would be expected to have similar results for the final migration rates. The slightly higher value in cationic peptides at pH 7, in both studies, could also be explained by another parameter such as the choice of the membrane in the EDUF configuration. In fact, Kadel et al. [21] recently demonstrated, using a well-characterized whey protein hydrolysate (0.75% (*w*/*v*) at pH 7), that there is a correlation between the physicochemical properties of filtration membranes and the global and individual peptide migration during EDUF. For the migration of peptides using PES 50 kDa membranes, the same membranes as in this study, Kadel et al. [21] observed that the global peptide migration rate in the cationic recovery compartment was higher with 3.12 ± 0.33 g/m^2^·h, compared to 2.92 ± 0.41 g/m^2^·h for anionic recovery compartment. At pH 7, the surface conductivity; zeta-potential, of the PES 50 kDa is negative (−13.4 ± 0.8 mV). This negative charge will attract positively charged peptides and favour their migration through the membrane. Therefore, the results in this study are totally in accordance with what was obtained in previous studies in terms of final peptide migration in EDUF.

### 3.2. Molecular Mass Distribution and Identification of Peptides in Recovery Compartments

**Figure 4:**
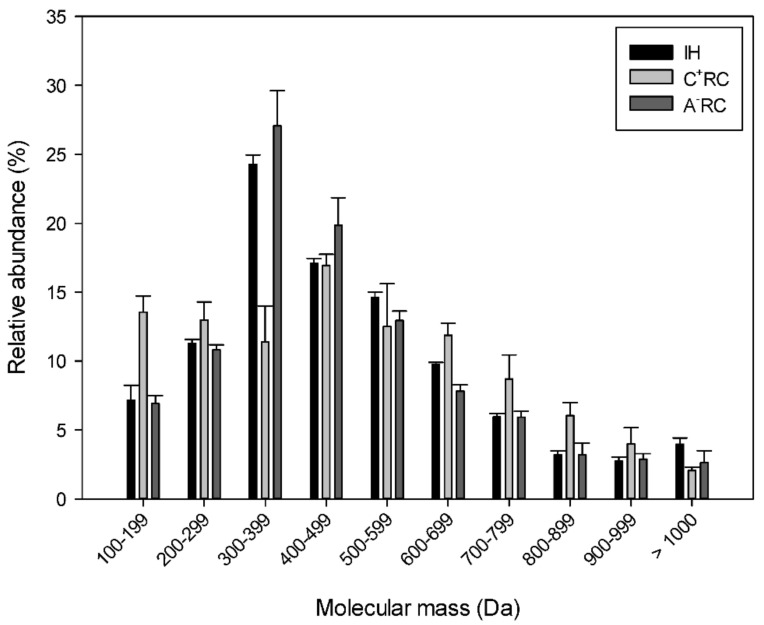
Relative molecular mass distribution in initial hydrolysate (IH), cationic (C^+^RC), and anionic (A^−^RC) recovery compartments after EDUF.

After injection of samples on UPLC-MS/MS, it was possible to extract the different compounds present in each recovery compartment. Out of these compounds, the relative molecular mass distribution (Figure 4) showed that the initial hydrolysate (IH) as well as both anionic and cationic recovery compartments contained mainly low mass peptides (<1000 Da) with a peak between 300 and 500 Da. Durand et al. [11] who worked with the same HMH, demonstrated, in the Supplementary Materials Section, that recovered fractions (anionic and cationic) at pH 6 had the same relative molecular mass distribution as for this study. There was a higher relative abundance of peptides in the small molecular masses range (300–500 Da), followed by a decrease as the molecular weight increased (Figure 4). Other studies on EDUF have displayed that, even when using different hydrolysate sources, small molecular weight peptides are recovered in the different recovery compartments [14]. Henaux et al. [10] studied EDUF applied to a salmon hydrolysate. The peptide molecular weight profiles clearly showed abundance in low mass peptides (<2000 Da) and a concentration, after the EDUF process, of peptides in the mass range of 300–600 Da. In the study by Kadel et al. [21] previously mentioned, the low mass peptides (<1200 Da) have also been found to migrate preferably through 50 kDa membranes. Hence, the results of this study confirm what was observed in previous studies even with different types of hydrolysates and smaller cell sizes.

**Figure 5:**
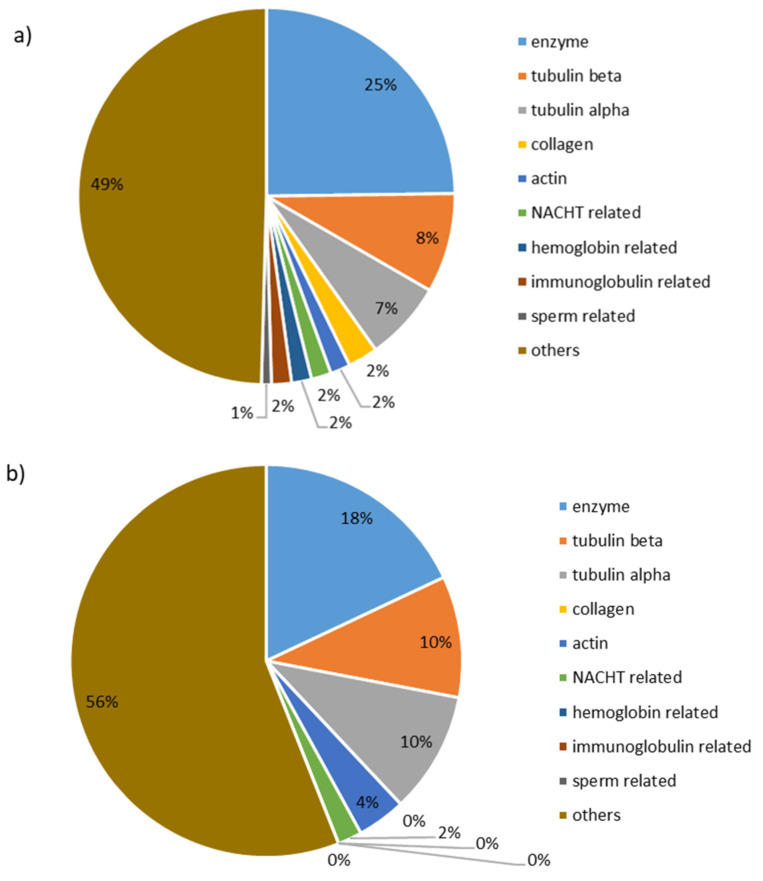
Origin identifications, by UPLC-MS/MS, of peptide sequences recovered after EDUF of herring milt hydrolysate in (**a**) anionic recovery compartment (A^−^RC) and (**b**) cationic recovery compartment (C^+^RC).

As for the identification of the sequence’s origins (Figure 5), despite a major part of “other” origins, “enzymes” are the main portion of the identifications with 25% and 18% for A^−^RC and C^+^RC, respectively. By combining the “alpha- and beta-tubulin” peptides, another large portion of the sequences is covered with 15% and 20% for A^−^RC and C^+^RC, respectively. It is very interesting to observe that the large portion of enzyme types would be due to the fact that enzymes are necessary for the catalysis of reactions in the herring embryo development. Tubulin alpha and beta are proteins that form microtubules, which serve as skeletal systems for living cells [22]. Actin plays an important role in eukaryotic cells where it has many crucial functions such as cell division and is a major constituent of the cytoskeleton [23]. Collagen, for its part, is a major structural element of all connective tissues and contributes to their stability and structural integrity [24]. Finally, there were sequences belonging to specific proteins that play a role in spermatogenesis (sperm-related peptide sequences). These are logical since the hydrolysate comes from milt which contains the genetic material for herring reproduction. Therefore, results observed in the present study can relate with peptide sequences obtained by using mass spectrometry and database search and with regard to the nature of the hydrolysate.

### 3.3. Areal System Resistance

**Figure 6:**
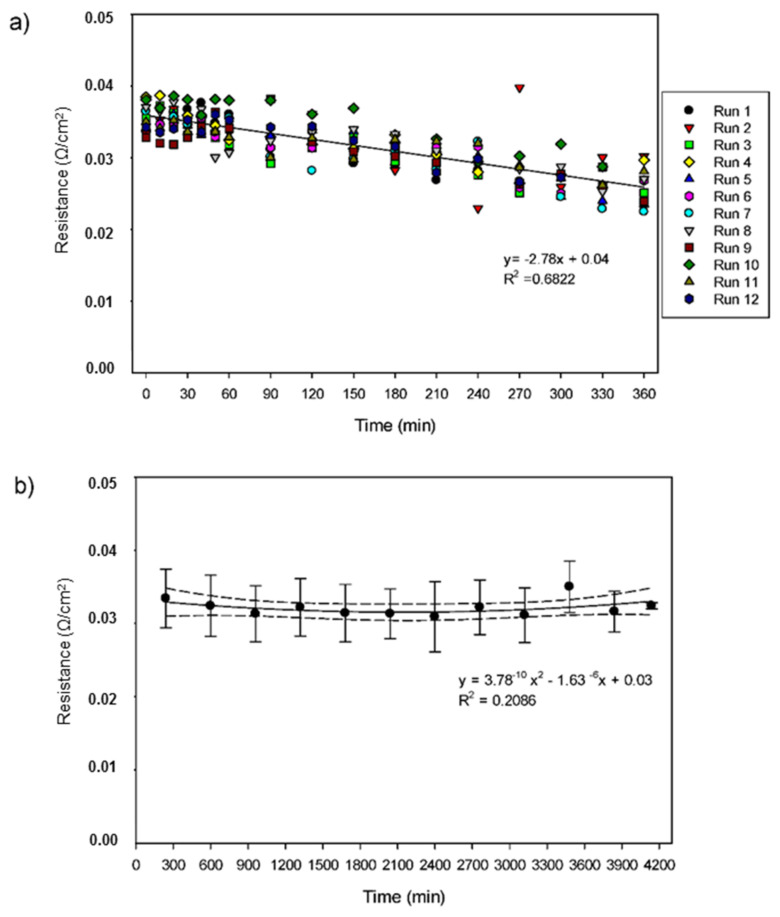
Evolution of the areal system resistance during EDUF process (**a**) for each of the 12 runs and (**b**) for the averaged 12 consecutive runs (4140 min). The dotted lines represent the 95% confidence interval.

This long-term study was performed in 12 experiments for a total of 69 h (4140 min) without dismantling the EDUF cell. The evolution of the global resistance during each run was relatively linear and varied from 0.0357 ± 0.0020 Ω/cm^2^ to 0.0263 ± 0.0027 Ω/cm^2^ (Figure 6a). Furthermore, the evolution of the averaged global resistance for 4140 min of total process duration slightly varied from 0.0309 ± 0.0048 to 0.0350 ± 0.0035 Ω/cm^2^, and values for each run were, except for run 10, found in the 95% confidence interval (Figure 6b). It appeared, from these results, that the resistance is quite stable and very reproducible in between experiments. In this case, with a 4% HMH, the highest concentration tested at this scale, a decrease in terms of resistance demonstrated that the process was not affected by fouling or by any demineralisation process since the conductivity was controlled during the runs. Effectively, the ions that migrated in a recovery compartment induced a decrease in electrical resistance in that compartment, and an increase in the initial compartment [7]. Therefore, the addition of salt ions (KCl) in the different compartments throughout the experiments, to maintain constant conductivity, allowed preventing such a phenomenon, thus explaining the slight decrease in the global electric resistance. Moreover, the stability of the averaged global system resistance was very interesting for such a long-term process considering the minimal cleaning performed in between runs. Persico et al. [17,18] studied the effect of peptide fouling on IEM and UF membranes and demonstrated that, with a salt solution, the ionic strength is high enough to allow the release of fouled peptides involved in electrostatic interactions with the membranes.

On the other hand, Suwal et al. [9] worked with a snow crab hydrolysate (2% *w*/*v*) for three runs of 6 h EDUF separations and demonstrated that keeping constant applied potential difference and conductivity in an EDUF system will keep the electric current of the system stable and allow quite linear migration. Koumfieg Noudou et al. [25] studied the effect of simultaneous peptide separation also from a snow crab hydrolysate at different start concentrations (three runs of 4 h for each concentration: 0.5, 1, 2, and 4%). It was also demonstrated that, during the EDUF treatment, the global system resistance remained constant (0.074 ± 0.0038 Ω/cm^2^) whatever the peptide concentration tested. This is explained by the constant potential difference applied and by maintaining the solution conductivities to their initial values throughout the experiments. In accordance with lab-scale studies, our study established the importance of maintaining these parameters constant to achieve constant global system resistance at the semi-pilot EDUF scale.

### 3.4. Relative Energy Consumption

**Figure 7:**
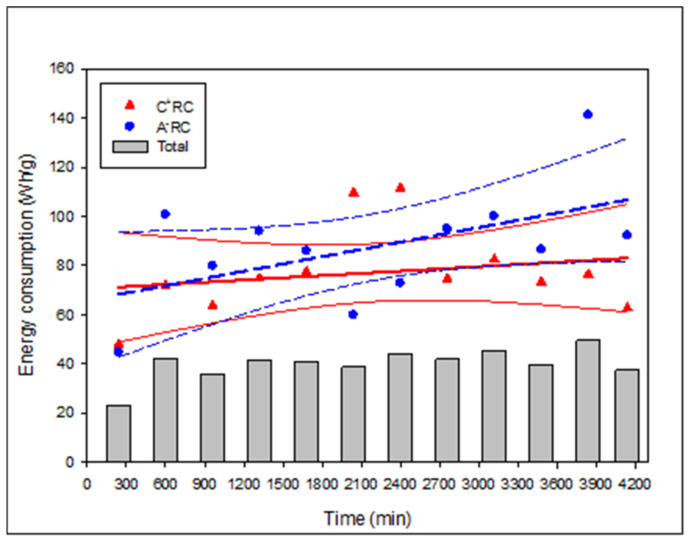
Evolution of the relative energy consumption for both cationic (C^+^RC) (full bold red line) and anionic (A^-^RC) (bold blue dotted line) recovery compartments as well as total energy consumption (bars) during EDUF process of 4140 min. The thin blue dotted lines and red full thin lines represent the 95% confidence interval for each recovery compartment.

The relative energy consumption (REC) calculated for each recovery compartment showed that the energy varied in between runs as much for C^+^RC as for A^−^RC, whereas looking at the total energy consumption, it was similar and constant at 39.95 ± 6.47 Wh/g for the 12 consecutive runs (Figure 7). For C^+^RC, REC varied from 47.94 to 111.55 Wh/g, while for A^−^RC, it fluctuated between 44.71 and 141.25 Wh/g. Considering the linear regression curves and the 95% confidence interval curves, the REC was quite constant for C^+^RC and would increase slightly for A^−^RC. Nevertheless, the 12-run averaged REC reveals that there is no significant difference (*p* = 0.232) between the energy consumption for the migration of anionic peptides (87.78 ± 23.76 Wh/g) and cationic peptides (77.20 ± 18.00 Wh/g). For C^+^RC, except for the first run, which was carried out for 4 h at a lower voltage (since this preliminary experiment was to set up the parameters), as well as runs 6 and 7, all the other REC values are in the 95% confidence interval. For A^−^RC, the rest of the data is within the 95% confidence interval except for runs 2, 6, 7, and 11. For runs 6 and 7, the high energy consumption for C^+^RC and the low energy consumption for A^−^RC can be explained by the fact that, in these runs, more anionic peptides were recovered (Figure 3), leading to less energy consumption for the anionic peptide’s migration than for the cationic peptides. As for runs 2 and 11 which are outside the 95% confidence interval with high energy consumption in A^−^RC, it could be due to system parameters that slightly fluctuated during the runs such as volume, temperature, or conductivity. In their study on another HMH, Durand et al. [12] observed that the total REC for migration of peptides was 46.99 ± 6.59 Wh/g. Since the configuration in the present study used eight UF membranes in EUR2-C type cell (4 × 50 kDa for each recovery compartment) instead of four in MP type cell for Durand et al. [12] (2 × 50 kDa and 2 × 20 kDa) which implies a larger membrane surface (800 cm^2^ vs. 100 cm^2^ per recovery compartment), it is conclusive to observe a higher REC value in this study. Additionally, Koumfieg Noudou et al. [25] studied the effect of a hydrolysate concentration (0.5, 1, 2, and 4%) on EDUF parameters. While also working with a MP-type cell (effective surface area of 100 cm^2^), they demonstrated that, at higher peptide concentrations, the REC values obtained were lower (3.53 Wh/g at 4% vs. 17.40 Wh/g at 0.5%). This is explained by the fact that there are more peptides available for migration in the recovery compartments; thus, the energy consumption required to migrate these peptides is lower [25]. Furthermore, the MWCO used for the membrane, which was high with 100 kDa, instead of 50 kDa in this study, allowed much more migration and offered less resistance [26]. Finally, in comparison with another type of configuration, using a MP-type cell, which contained three UF membranes of different MWCO for migration of either cationic or anionic peptides, Henaux et al. [10] obtained, with 6 h runs for a salmon protein hydrolysate (pH 6, 0.7% (*w*/*v*)), very high REC of 512.56 ± 95.59 Wh/g and 849.71 ± 80.18 Wh/g for cationic and anionic peptides, respectively. Firstly, the low concentration hydrolysate will display higher REC, but also the stacking of three different MWCO membranes will require more energy to allow peptides to migrate through all the compartments. This demonstrates, again, that the cell and configuration used in this study present quite acceptable REC. Additionally, the fact that, from run to run, it was possible to recover the initial current and voltage values and keep REC stable, could indicate that the salt cleaning after each treatment seems to be enough to re-establish the initial working conditions after each run.

### 3.5. Membrane Characterization

#### 3.5.1. Thickness

**Figure 8:**
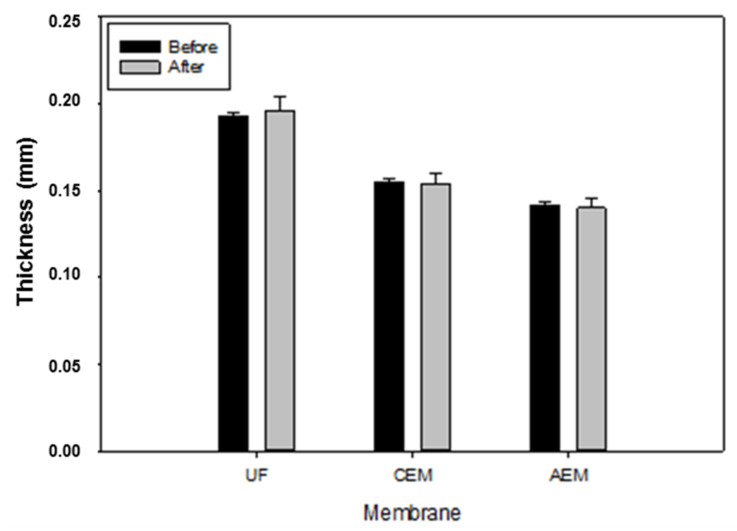
Average thickness of ultrafiltration (UF), cationic (CEM), and anionic (AEM) membranes before and after the EDUF process. t-test was performed at *p* ≤ 0.05.

As it is now common knowledge, changes in the ultrafiltration membrane conductivity and thickness are effective indicators to evaluate membrane fouling during the EDUF process [13]. Therefore, the overall average thickness values showed no significant differences before and after the EDUF process after t-test statistical analysis (*p* ≤ 0.05) for the three types of membranes (Figure 8). Indeed, for UF membranes the thickness was 0.193 ± 0.002 mm before and 0.196 ± 0.008 mm after EDUF. In comparison, AEM had values of 0.142 ± 0.002 mm and 0.140 ± 0.006 mm before and after the entire EDUF process, respectively. Finally, for the only CEM present in the configuration, its thickness values were 0.155 ± 0.002 mm before and 0.154 ± 0.006 mm after the runs. In another study, Doyen et al. [27] observed no significant differences of thickness for UF PES 20 kDa membranes after 54 h of EDUF treatment with a snow crab hydrolysate by doing regular cleaning-in-place (CIP) with industrial chemical solutions after each experiment. More recently, Persico et al. [17,18] demonstrated, using a whey protein hydrolysate that the main peptide fouling found on ion-exchange membranes and UF membranes was caused by electrostatic interactions that can be broken by changing the ionic strength, thus the use of a 2% NaCl solution. Additionally, in their study on UF membranes, Persico et al. [18] demonstrated that PES membrane material showed very negligible peptide fouling, which is the material used in this study. As discussed previously for REC values, regarding these results, it appeared that the salt cleaning solution after each experiment allows removing potential fouling on the different membranes. No fouling was observed on any of the membranes when the stack was opened for inspection. Finally, the thickness values obtained in this study for UF PES 50 kDa membranes are constant from one lot to another since Kadel et al. [21] measured a value of 0.203 ± 0.004 mm thickness for the same membrane type, while the value obtained in this study is 0.193 ± 0.002 mm.

#### 3.5.2. Conductivity

**Figure 9:**
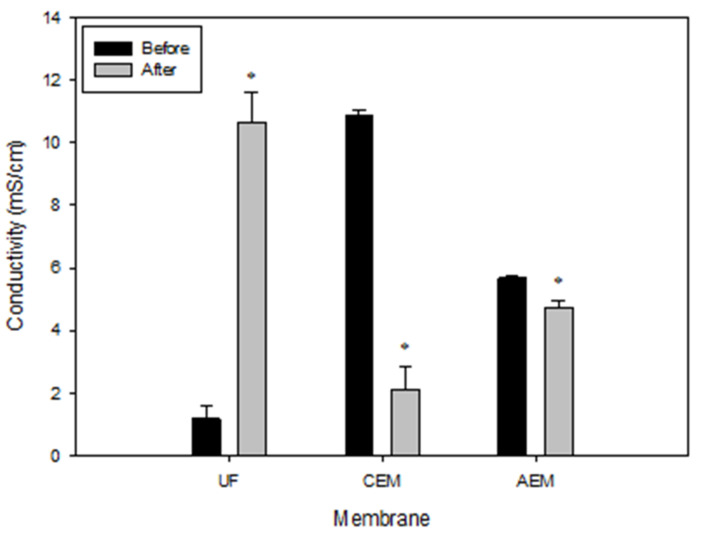
Average conductivity for ultrafiltration (UF), cationic (CEM), and anionic (AEM) membranes before and after the EDUF process. T-test was performed at *p* ≤ 0.05. * over a band means a significant difference.

The average conductivity of the three types of membranes is displayed in Figure 9. It can be observed that UF membranes exhibit a significant difference (*p* ≤ 0.05) before and after the EDUF process with values of 1.189 ± 0.383 mS/cm before and 10.657 ± 0.954 mS/cm after. As for the AEMs, the average values show a small but significant (*p* ≤ 0.05), decrease in conductivity after the EDUF process, varying from 5.66 ± 0.07 mS/cm before to 4.74 ± 0.2 mS/cm after. The average conductivity also decreased for the only CEM in the stack with values of 10.85 ± 0.17 to 2.1 ± 0.75 mS/cm for before and after, respectively. The conductivities of the ion exchange membranes before the EDUF process compared with the expected values from the literature since, as shown in other studies, the conductivity before EDUF of the AEM or CEM were of similar values around 8–9 mS/cm for AEM and 10–11 mS/cm for CEM [17,28]. The large increase in conductivity for the UF membranes may be explained by the fact that they are received dried from the manufacturer. They must be presoaked in a saline solution prior to being used in EDUF. Thus, this set of membranes was soaked for a short period of time before being stacked in the EDUF cell. As the experiments were carried out, using saline solutions acted as a form of conditioning for the UF membranes, and this has a major impact on the electrical conductivity value of an ultrafiltration membrane [29]. For other experiments in our lab (data not shown), some of the same UF membranes were soaked in saline solution for about three months, and the conductivity measurement gave a value of around 10.9 mS/cm. This confirms the conditioning of UF membranes over time with saline solution.

For the ion-exchange membranes, it could be expected that after 69 h of process, the effectiveness would be affected since many studies have demonstrated and explained the fouling phenomena which may occur on these types of membranes [30,31]. In Figure 9, it is possible to observe that the CEM had a much lower conductivity recovery than the AEM after salt solution cleaning. This may be explained by Langevin and Bazinet [32], who demonstrated that the impact of NaCl solution to remove peptides fouled on AEM was more pronounced than for CEM. Moreover, the CEMs were almost two times more sensitive to peptide fouling than AEMs. Furthermore, the peptide species and nature of peptide/AEMs interactions involved in fouling were explored at different pH (2, 6, 10) by Persico et al. [17]. Using a well-characterised whey hydrolysate, they illustrated that, as the pH of the hydrolysate increased, more peptide fouling occurred on AEM. It was explained that the more crucial parameter was the peptide charge which depends on its amino acid composition. The same team also did a similar study but this time using CEM [33]. It was revealed that the most fouling occurred at pH 6 when pH 2 was expected to have the most fouling. The explanation given was regarding the structure of the membrane at pH 2 in which, part of the sulphonic groups (SO_3_H/SO^3−^) on the CEM, would be protonated, thus preventing electrostatic interactions. Another possible hypothesis is that, at pH 6, peptide/peptide hydrophobic interactions would increase the presence of peptides on the CEM compared to pH 2. Since in this study, the pH of the HMH was adjusted to 7, these deductions may bring to light why the CEM’s conductivity has decreased more than for the AEMs. On another note, it has been demonstrated that free amino acids can play an important role in the fouling of ionic membranes. Sandeaux et al. [34] studied the presence of free arginine (a basic amino acid which bears a positive electrical charge at neutral pH) and its effect on membrane fouling and transport properties using model solutions. They specifically studied its effect on cation-exchange membranes. This could help explain why the only CEM in our configuration had such a low conductivity after 69 h of process. Suwal et al. [28] studied the effect of free amino acids on ion-exchange membranes and UF membranes (PES 20 kDa) before and after six successive peptide fractionations of 6 h (36 h total). They used a 2% (*w*/*v*) snow crab hydrolysate at pH 6 and the amino acid content revealed the highest amount of free amino acid was for arginine, with 4.79 ± 0.01 g/100 g. This is three times lower than for the HMH in this study which has an arginine concentration of 15.38 ± 0.97 g/100 g [11], but still, they observed a significant decrease in membranes conductivity after six runs. In fact, the AEM had a value of 8.92 ± 0.35 mS/cm before and a value of 0.76 ± 0.05 mS/cm after the treatments. For the CEM, they both had a value around 10.82 ± 0.18 mS/cm before and decreased around 4.76 ± 0.8 mS/cm after treatments. Finally, for the UF membranes, there was no significant difference between before and after the six runs (5.30 ± 0.52 vs. 5.85 ± 0.19 mS/cm). The results observed in this study for IEM and UF membranes are in accordance with the previous results obtained in the different studies on pH of the hydrolysate, fouling by amino acids, and conditioning of the UF membranes throughout the EDUF experiments with saline solutions. Consequently, according to the evolution of conductivity and thickness values, no fouling was observed on the UF membranes, indicating the integrity and efficiency were not affected after a total of 69 h of HMH separation by EDUF. Finally, the values obtained for the IEM in the stack are in accordance with the different possible fouling phenomena described in the literature.

## 4. Conclusions

According to these results, we demonstrate, for the first time, the feasibility of a long-term EDUF process to recover bioactive peptide fractions with simple but effective salt solution cleaning in between experiments. First, the peptide migration showed specificity and reproducibility throughout the 12 consecutive experiments. As for final migration rate, the values obtained for A^−^RC and C^+^RC can relate to other studies and demonstrated very interesting rates (5.2 ± 0.8 g/m^2^·h for C^+^RC and 4.7 ± 1.1 g/m^2^·h for A^−^RC) even if the percentage of peptides separated (3.77%) were low. However, as demonstrated in many previous studies, the biological activity is increased or appeared, in some cases, after EDUF, and high specificity of the separated peptides, in comparison with other processes, is put forward. Some parameters could be modified to obtain more quantity of peptides if needed such as duration, voltage, or intensity. The molecular mass distribution showed that the recuperation fractions were composed of low molecular mass peptides (<1000 Da), and the identification of the sequences confirmed that they are in accordance with the hydrolysate’s origin. Since the global system resistance slightly decreased within a run and was quite stable throughout the 69 h of treatment, we claim that the salt solution cleaning in between runs is suitable for removing peptides that may have fouled on the membranes. The same interpretation can be used to explain the almost constant and low relative energy consumption (39.95 ± 6.47 Wh/g).

Since the pH of the hydrolysate was kept constant at 7, the peptide migration was quite similar in both recovery compartments. In future experiments, it could be considered to adjust the hydrolysate’s pH at a preferred value if either cationic or anionic peptides need to be recovered to maximise the bioactivity potential of a fraction. Besides, different configurations could be tested to optimise peptide recovery and process parameters. In addition, for the present hydrolysate, it is worth separating cationic and anionic peptides because they display different bioactivities. In a possible future industrial process, it could be interesting to pool some of the fractions together to see if the bioactivities are increased. Following these results, a scale-up study is currently being carried out in our laboratory on a preindustrial EUR6 EDUF cell (Ameridia). The achievements brought by this long-term EDUF process pave the way to pursue the development of EDUF processes at larger scales and, eventually, have working EDUF systems in the food industries to produce bioactive peptides or for the separation of other charged molecules of interest.

## Figures and Tables

**Table 1:** Comparison of previous pilot-scale (EUR2-C stack) studies for the EDUF separation of different hydrolysates.

Reference	Hydrolysate; Concentration; pH; Volume	# of UF Membrane; Membrane Surface	Membrane Type	Number of Experiments	Duration of Experiments/ Total Time	ED Parameters	Final Migration Rate (g/m^2^·h)	Cleaning
Firdaous et al. [13]	Alfalfa white; 0.5% (*w*/*v*); pH 3 and 9; 3 L	6 UF total; 0.12 m^2^	PES 10 kDa	3 P− and 3 P+	120 min/ 720 min	8 V	P−: 5.3 P+: 8.7	CIP of the system only, not stack, with alkaline and acid solutions
Langevin et al. [15]	Soy; 0.1% (*w*/*v*); pH 3, 6, 9; 1.5 L	8 UF total; 0.08 m^2^/RC	PES 10 kDa	3 simultaneous P− and P+ for each pH	180 min/ 1620 min	8 V	P−: pH 3 = 0.109 pH 9 = 0.165 P+: pH 3 = 0.238 pH 9 = 0.097	*n*/*m*
Doyen et al. [14]	Snow crab; 1% (*w*/*v*); pH 9; 2.5 L	6 UF total; 0.06 m^2^/RC	PES 20 kDa	4 simultaneous P− and P+ for each voltage	60 min/ 960 min	2, 4, 6 and 8 V	6.67	CIP after each run with Ecolab cleaning solutions *, then stack dismantled
Roblet et al. [16]	Soy; 2.4% (*w*/*v*); pH 3, 6, 9; 2 L	6 UF total; 0.06 m^2^/RC	PES 10 kDa	3 simultaneous P− and P+ for each pH	240 min/ 2160 min	5 V	P−: 1.3 P+: 2.4	CIP after each run with Ecolab cleaning solutions *, then stack dismantled
This study	Herring milt; 4% (*w*/*v*); pH 7; 3 L	8 UF total; 0.08 m^2^/RC	PES 50 kDa	12 simultaneous P− and P+	240–360 min/ 4140 min	14 V	P−: 4.7 P+: 5.2	Sodium chloride (NaCl) 2% solution

RC = recovery compartment; P+ = cationic peptides; P− = anionic peptides; *n/m* = not mentioned; CIP: cleaning in place. * Ecolab cleaning solutions consist of 2% Membra-Chlore 310 (basic cleaning with sodium hydroxide and chlorine) and 1% Ultrasil 75 (acid cleaning with phosphoric acid).

## Data Availability

Not applicable.

## Supplementary Materials

The following are available online at https://www.mdpi.com/article/10.3390/membranes11080558/s1, Figure S1: Thickness of ultrafiltration membranes used in the EDUF cell before and after the entire 69 h process. T-test was performed at *p* ≤ 0.05. * over a bar means a significant difference in comparison with its respective membrane before EDUF, Figure S2: Thickness of anion-exchange membranes (AEM) used in the EDUF cell before and after the entire 69 h process. T-test was performed at *p* ≤ 0.05. * over a bar means a significant difference in comparison with its respective membrane before EDUF, Figure S3: Conductivity of all ultrafiltration membranes used in the EDUF cell before and after EDUF process. t-test was performed at *p* ≤ 0.05. * over a band means a significant difference, Figure S4: Conductivity of the four anion-exchange membranes (AEM) used in the EDUF cell before and after the EDUF process. t-test was performed at *p* ≤ 0.05. * over a band means a significant difference.
