# Supplementary material for: Scale-Up and Long-Term Study of Electrodialysis with Ultrafiltration Membrane for the Separation of a Herring Milt Hydrolysate
Source: Membranes (Basel). 2021 Jul 23;11(8):558. doi: 10.3390/membranes11080558 (PMC8399119; doi:10.3390/membranes11080558)
Supplement: Supplementary file 1 [file membranes-11-00558-s001.zip › membranes-1178244-supplementary.pdf]

membranes

Supplementary information

Scale-Up and Long-Term Study of Electrodialysis with Ultrafiltration Membrane for the Separation of a Herring Milt Hydrolysate

Jacinthe Thibodeau, Noémie Benoit, Véronique Perreault, and Laurent Bazinet *

Laboratoire de Tranformation Alimentaire et Procédés ÉlectroMembranaires (LTAPEM, Laboratory of Food Processing and Electromembrane Process), Department of Food Sciences, Institute of Nutrition and Functional Foods (INAF), Université Laval, Québec, QC G1V 0A6, Canada; Jacinthe.thibodeau.1@ulaval.ca (J.T.); Noemie.benoit.1@ulaval.ca (N.B.); Veronique.perreault.5@ulaval.ca (V.P.)

* Correspondence: Laurent.bazinet@fsaa.ulaval.ca; Tel.: +1-418-656-2131 (ext. 407445); Fax: +1-418-656-3353

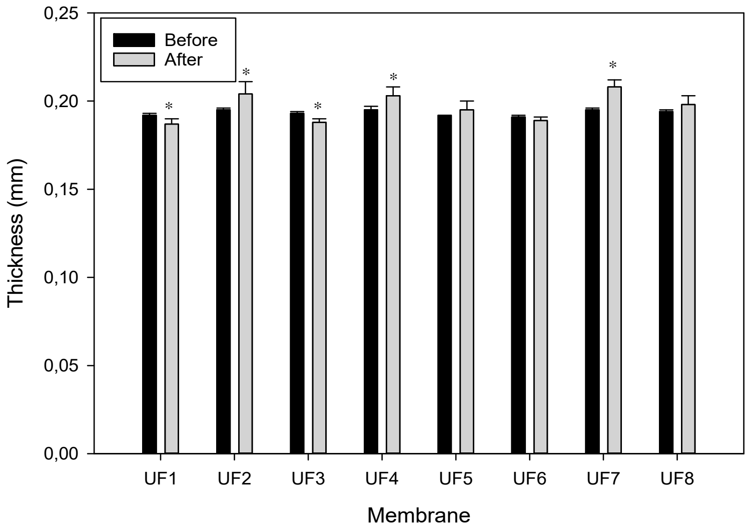

Figure S1. Thickness of ultrafiltration membranes used in the EDUF cell before and after the entire 69-hour process. t-test was performed at $p \leq 0.05$. * over a bar means a significant difference in comparison with its respective membrane before EDUF.

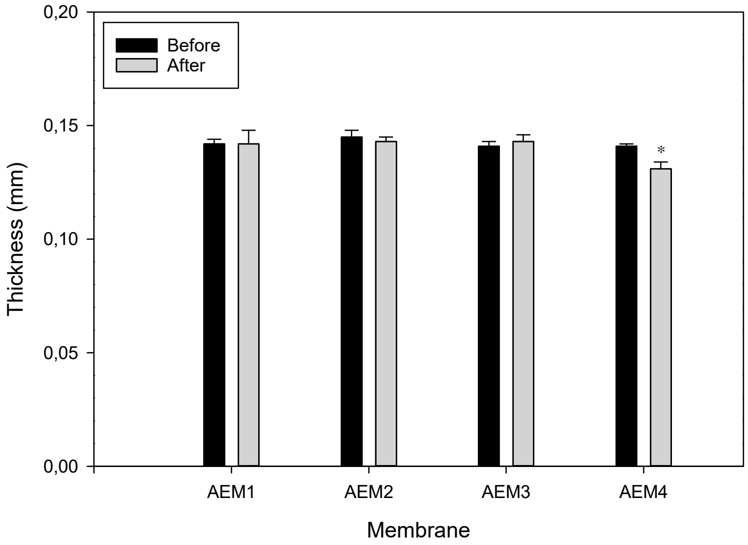

Figure S2. Thickness of anion-exchange membranes (AEM) used in the EDUF cell before and after the entire 69-hour process. t-test was performed at $p \leq 0.05$. * over a bar means a significant difference in comparison with its respective membrane before EDUF.

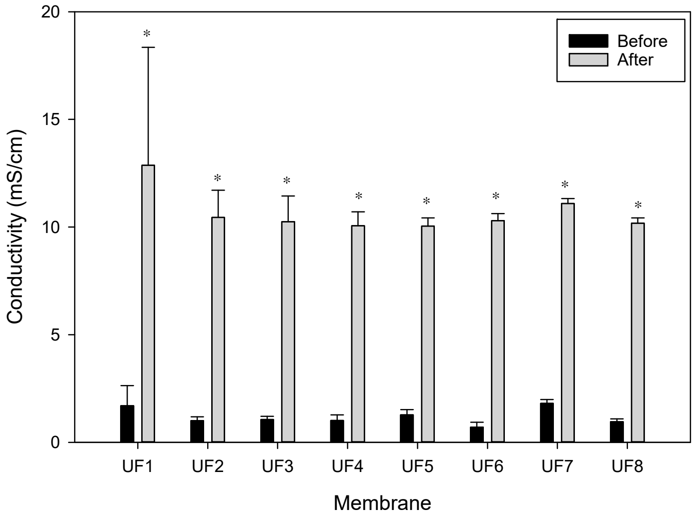

membranes

Figure S3. Conductivity of all ultrafiltration membranes used in the EDUF cell before and after EDUF process. t-test was performed at $p \leq 0.05$. * over a band means a significant difference.

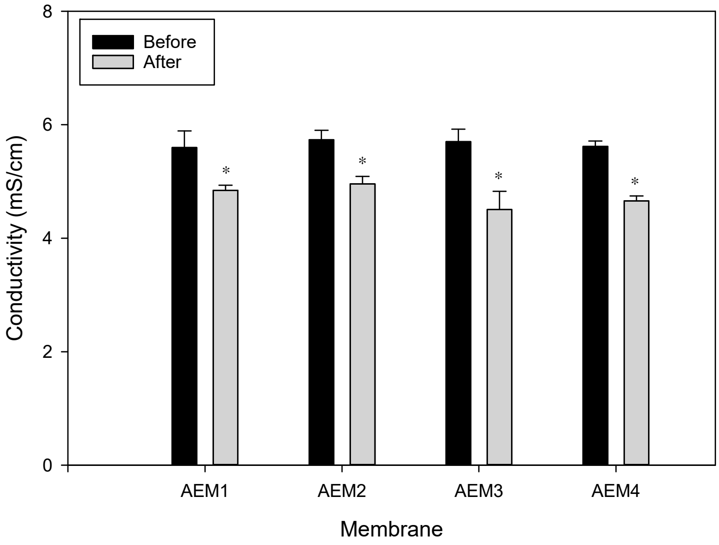

Figure S4. Conductivity of the four anion-exchange membranes (AEM) used in the EDUF cell before and after EDUF process. t-test was performed at $p \leq 0.05$. * over a band means a significant difference.
